# Correction: Induced TRPC1 expression sensitizes intestinal epithelial cells to apoptosis by inhibiting NF-κB activation through Ca2+ influx

**DOI:** 10.1042/BCJ20060124_COR

**Published:** 2026-05-21

**Authors:** Jian-Ying Wang, Bernard S. Marasa, Jaladanki N. Rao, Tongtong Zou, Lan Liu, Kaspar M. Keledjian, Ai-hong Zhang, Lan Xiao, Jie Chen, Douglas J. Turner

**Affiliations:** Department of Pathology, University of Maryland, School of Medicine, Baltimore, Maryland 21201, U.S.A.

**Keywords:** capacitative calcium entry (CCE) mechanism, IκB, mucosal homoeostasis, polyamine, programmed cell death, store-operated Ca2+ channel (SOC)

The authors of the original article “Induced TRPC1 expression sensitizes intestinal epithelial cells to apoptosis by inhibiting NF-κB activation through Ca2+ influx” (DOI: 10.1042/BJ20060124) would like to correct Figure 3A. Several similarities between Figures 1B 3A and were noted by a reader, which included:

- The Figure 1B No-TNF/CHX/TRPC1 Cells panel and the Figure 3A No-Staurosporine/IEC-6 Cells panel
- The Figure 1B TNF/CHX 2hr/TRPC1 Cells panel and the Figure 3A Staurosporine 1 h/IEC-6 cells panel
- The Figure 1B TNF/CHX 4 hr/IEC-6 Cells panel and the Figure 3A Staurosporine 2 hr/IEC-6 cells panel

The authors explained that the Figure 1B images were correct, and that some of these images were mistakenly used in the final figure preparation of Figure 3A. They were also able to provide raw data. The correction has been assessed and approved by the Editorial Board. The authors apologise for this error and declare that these corrections do not change the results or conclusions of their paper.

The corrected version of Figure 3A is presented here:

**Figure 3A:**
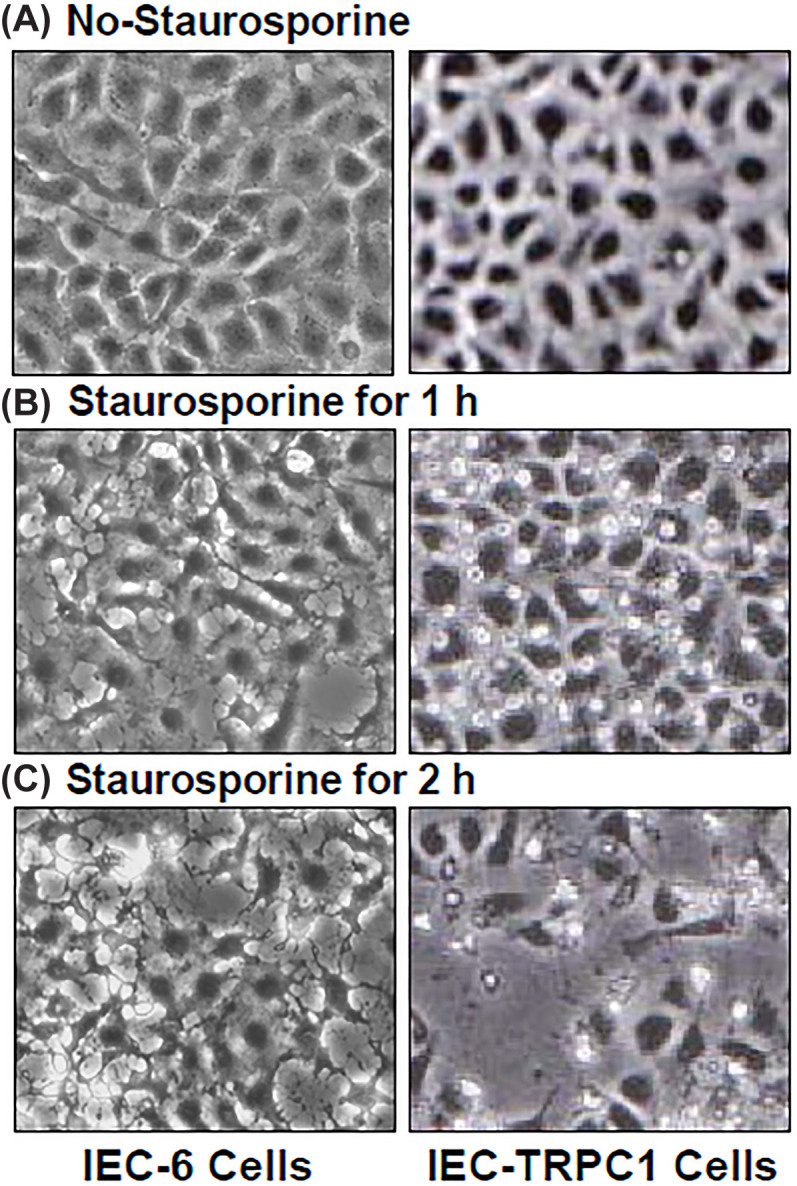
Images of staurosporine (STS)-induced apoptosis in control IEC-6 cells (*left panels*) and stable IEC-TRPC1 cells (*right panels*) (**A**) cells treated without STS; (**B**) cells treated with STS for 1 h; and (**C**) cells treated with STS for 2 h. Original magnificent X150. Bernard S. Marasa et al., *Biochem J* (2006)

